# Isolated Blind-Ended Major Aortic Pulmonary Collateral Artery With an Aneurysm in an Infant With Trisomy 21

**DOI:** 10.7759/cureus.72078

**Published:** 2024-10-21

**Authors:** Ryota Yonaga, Yoshihiko Kodama, Kazunari Takamura, Masako Harada, Hiroshi Moritake

**Affiliations:** 1 Division of Pediatrics, Faculty of Medicine, University of Miyazaki, Miyazaki, JPN

**Keywords:** aortopulmonary collateral artery, down's syndrome, interventional congenital cardiologist, pediatric congenital heart disease, vascular plug

## Abstract

In infants, anomalous vessels from the aortic arch are often diagnosed as patent ductus arteriosus or bronchial arteries. We present the case of a 22-day-old female with trisomy 21, found to have a blind-ended aneurysmal vessel originating from the descending aorta. Echocardiography revealed bidirectional flow and computed tomography confirmed the aneurysm without peripheral vessel connection. Since the vessel was not connected to the pulmonary artery and did not course along the peripheral bronchi, we diagnosed the vessel as a blind-ended major aortopulmonary collateral artery. The vessel was treated with catheter embolization using an Amplatzer vascular plug (Abbott Laboratories, Chicago, IL, USA).

## Introduction

In infants, echocardiographic studies sometimes show a vessel arising from the lesser curvature of the aortic arch. Such a vessel is frequently diagnosed as either a patent ductus arteriosus or a bronchial artery that supplies blood flow to the bronchus [1]. A major aortopulmonary collateral artery (MAPCA) is an anomalous vessel that supplies blood flow to the peripheral pulmonary arteries, and it is typically observed in cases of compromised pulmonary circulation such as pulmonary atresia and severe pulmonary stenosis. In such cases, MAPCAs are required to be surgically unifocalized to one blood flow system from the ventricle [2]. There have been reports of cases in which MAPCAs are isolated congenital heart disease without pulmonary atresia or stenosis [3], which can sometimes complicate aneurysm [4]. We present a case report of blind-ended isolated MAPCA with an aneurysm, which was successfully treated by catheter intervention.

## Case presentation

**Figure 1:**
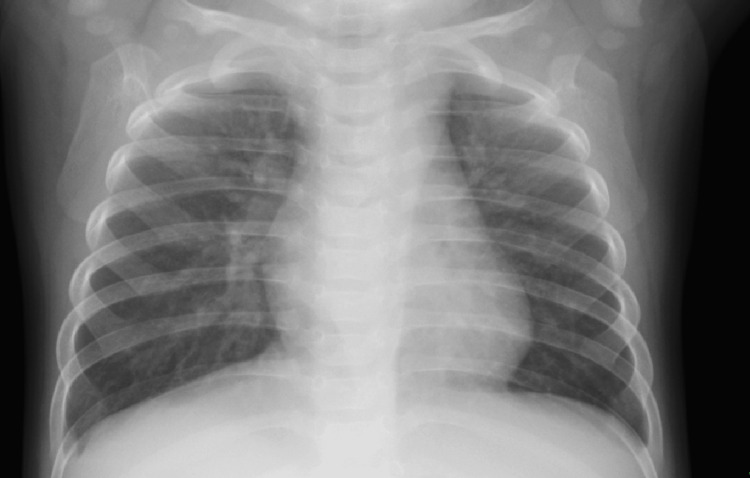
Chest radiography Chest radiography showed no cardiomegaly.

**Figure 2:**
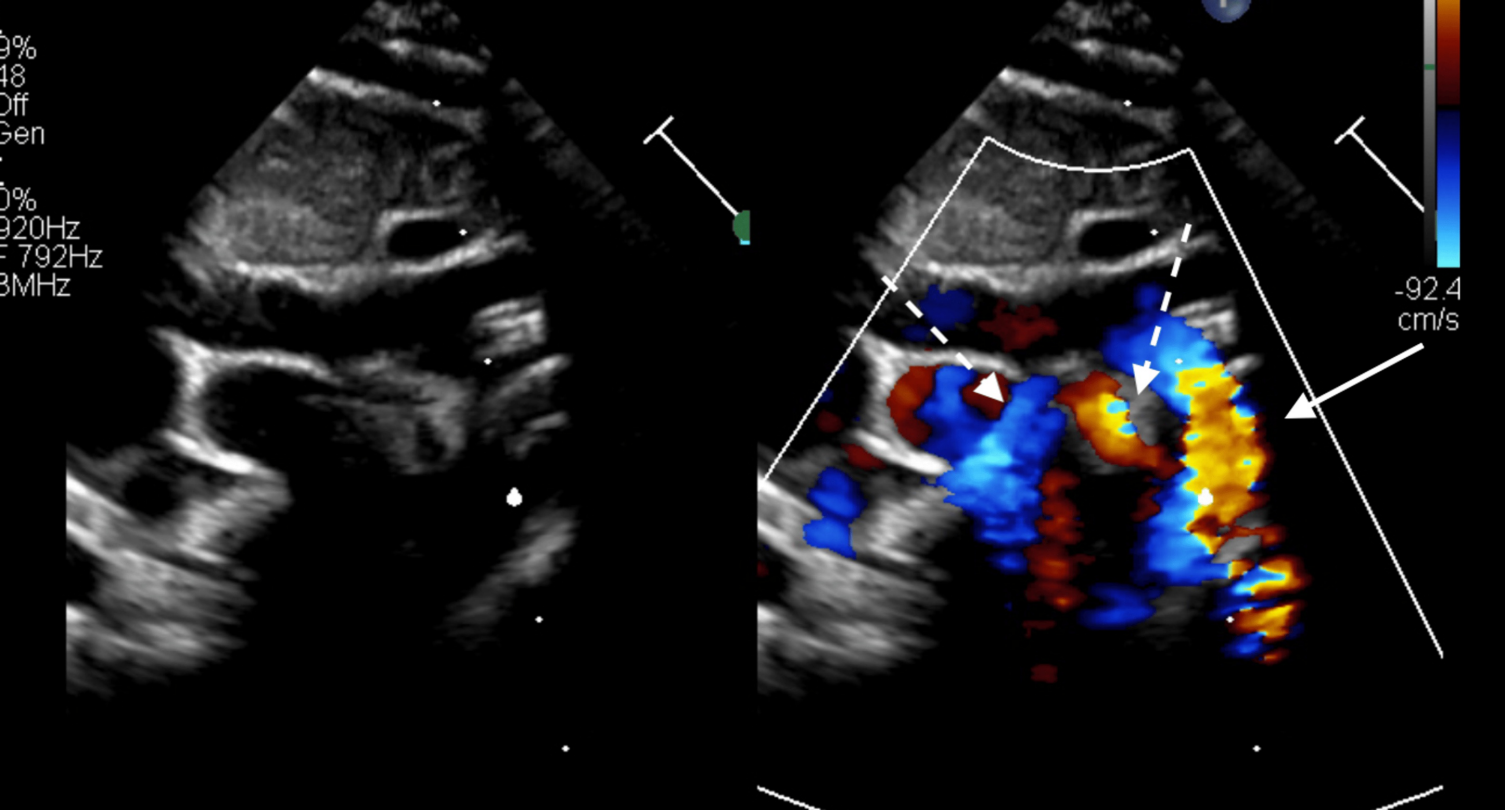
Echocardiography (arch view) Echocardiography showed an abnormal vessel originating from the descending aorta. The solid arrow indicates the aortic arch and the dashed arrow indicates the abnormal vessel.

**Figure 3:**
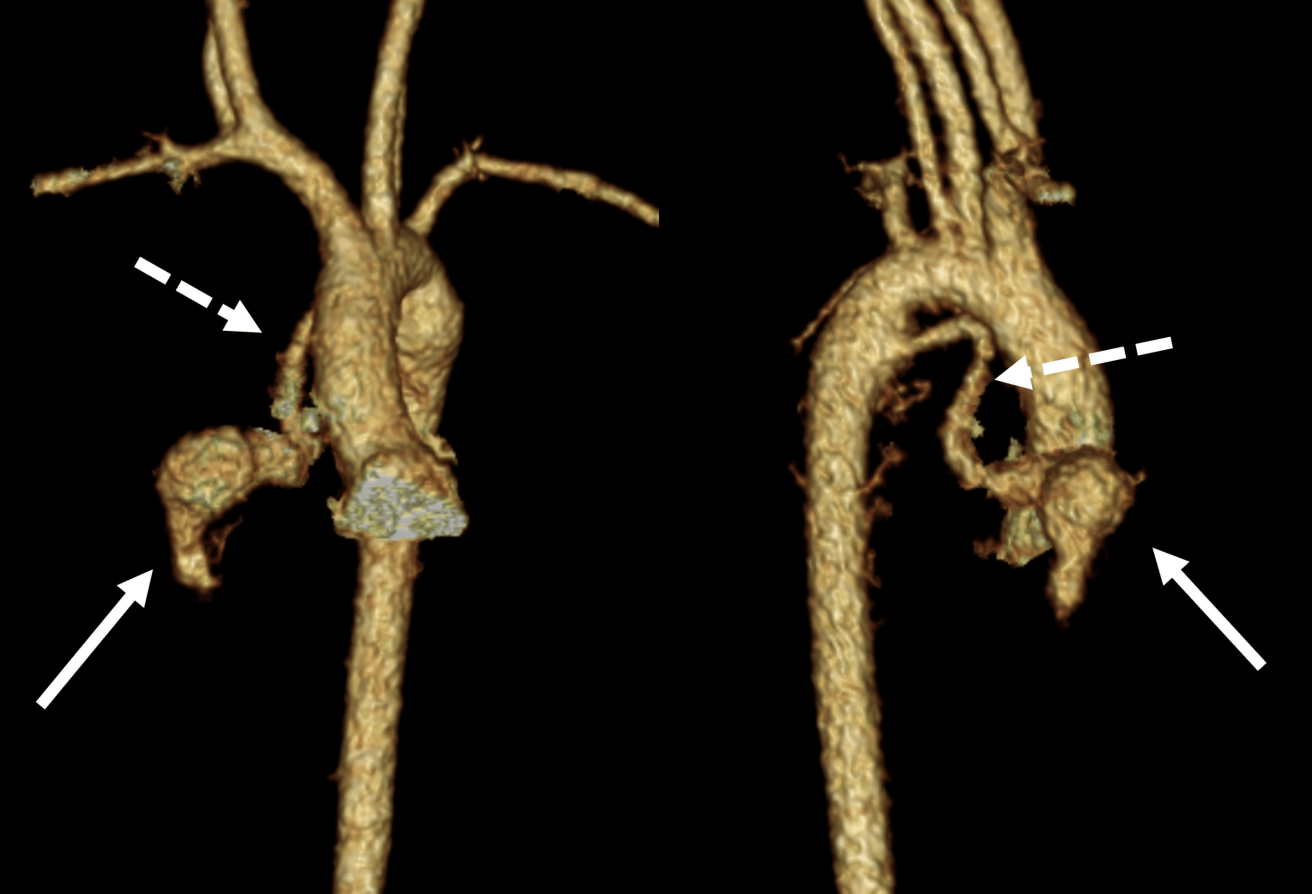
Contrast-enhanced computed tomography (three-dimensional reconstructed image) Contrast-enhanced computed tomography (three-dimensional reconstructed image) demonstrated an abnormal vessel branching off the descending aorta with an associated aneurysm. The arrow indicates an aneurysm and the dashed arrow indicates an abnormal vessel.

**Figure 4:**
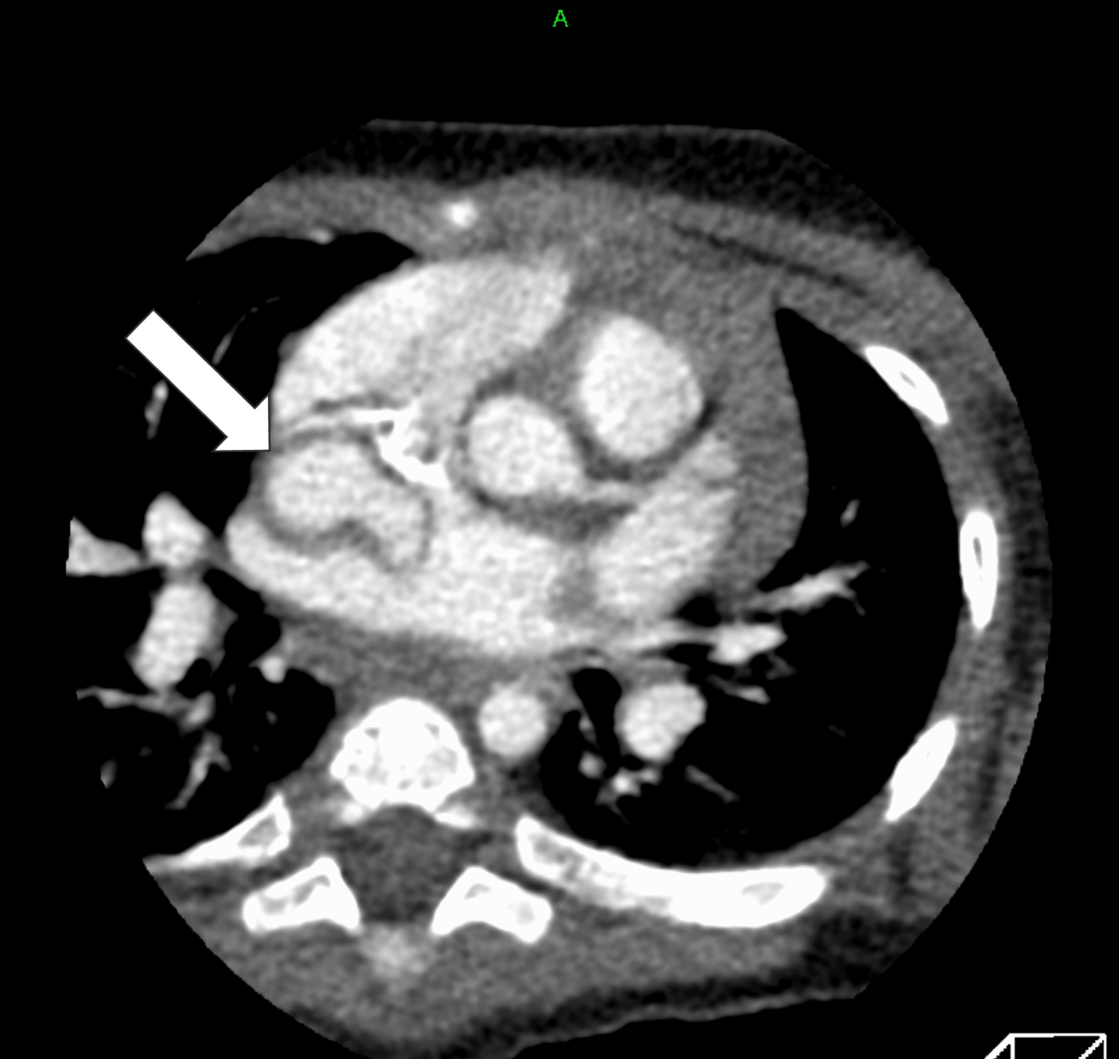
Contrast-enhanced computed tomography (axial image) Contrast-enhanced computed tomography (axial image) demonstrated an aneurysm between the right atrium and the right pulmonary vein.

**Figure 5:**
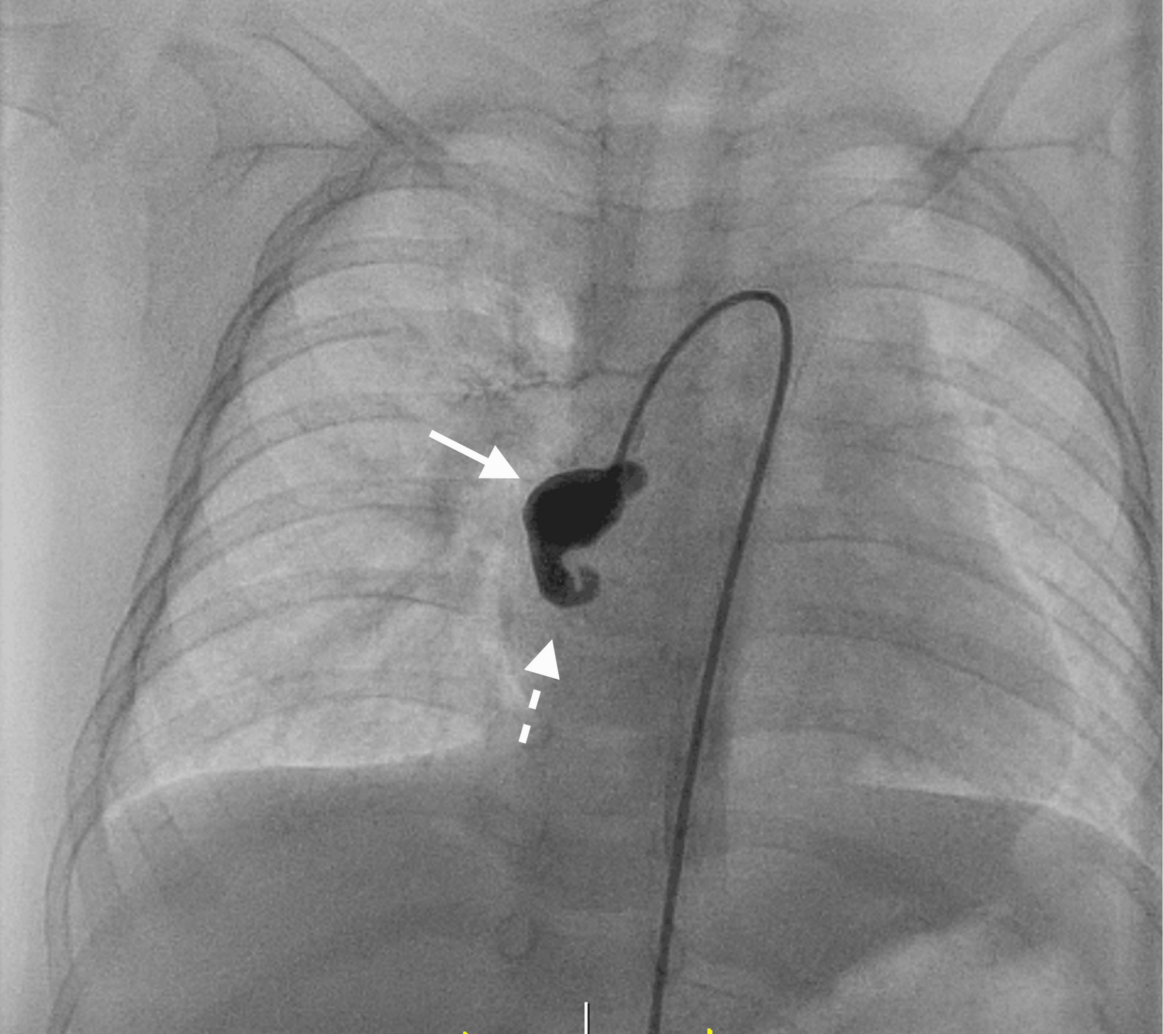
Angiography The arrow indicates an aneurysm. The dashed arrow indicates the blind end of the vessel.

**Figure 6:**
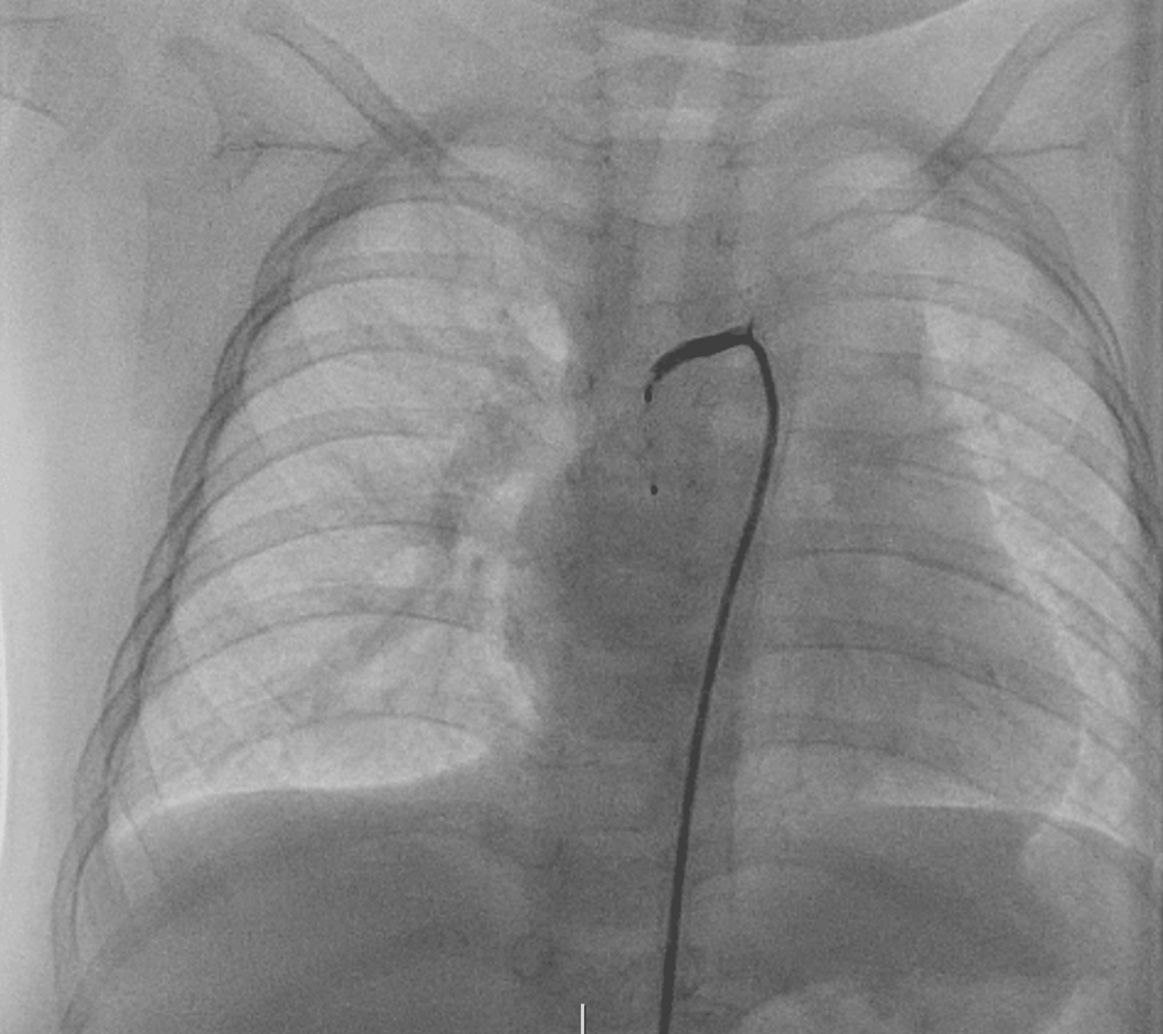
Angiography after embolization Angiography after embolization showed complete occlusion of the vessel.

A 22-day-old female infant previously diagnosed with trisomy 21 presented to our hospital for routine cardiac assessment. She was born weighing 2177 g at 39 weeks gestational age and had an oxygen saturation of 99% on room air. She had no symptoms of heart failure, and chest radiography showed no cardiomegaly (Figure 1). Echocardiography revealed no intracardiac structural abnormalities except for a patent foramen ovale and an abnormal vessel originating from the descending aorta (Figure 2). Blood flow through the vessel was bidirectional, but the peripheral side of the vessel could not be identified. Contrast-enhanced computed tomography showed an aneurysm on the abnormal vessel, which is located between the right atrium and the right pulmonary vein; continuity with another vessel was not shown (Figures 3, 4). No change in vessel size was observed on an echocardiogram in the subsequent period. Cardiac catheterization was performed at age eight months with a body weight of 6.5 kg. Pressure measurement did not show pulmonary hypertension (pulmonary artery pressure 22/9/mean 16 mmHg) and there was no decrease in diastolic aortic pressure (aortic pressure 92/52/mean 71 mmHg). Angiography showed the anomalous vessel was blind-ended and had an aneurysm; blood flow through the vessel was bidirectional (Figure 5). Considering the risks of aneurysm rupture and thrombus formation, we then performed occlusion of the vessel using a 4 × 10 mm Amplatzer vascular plug 4 (Abbott Laboratories, Chicago, IL, USA). We planned to place the plug in the area where the vessels run in a straight line, and the procedure was performed safely with no adverse events. Post-embolization angiography showed complete occlusion (Figure 6).

## Discussion

Vessels seen branching off the descending aorta in infancy are, in most cases, either a ductus arteriosus, bronchial artery, or MAPCA. The ductus arteriosus connects the aorta to the pulmonary artery while the bronchial artery branches off the aorta and courses along the peripheral bronchi. MAPCAs provide pulmonary blood flow and are usually associated with congenital heart disease which is associated with an absence of a main pulmonary artery originating from the ventricle. Embryologically, during early fetal development, the vascular plexus in the lung buds is connected to the segmental arteries arising from the dorsal aorta. If pulmonary blood flow is poor because of pulmonary atresia, the segmental arteries remain as MAPCAs [5]. However, there are reports of isolated MAPCA without congenital heart disease, which usually regresses spontaneously [3]. In our patient, the abnormal vessel exhibited bidirectional flow on echocardiography and no peripherally connecting vessel was identified. Contrast-enhanced computed tomography also failed to identify the peripheral connecting vessels, and an abnormal vessel was found to have an aneurysm between the left atrium and pulmonary vein. As the vessel was not connected to the pulmonary artery and did not course along the peripheral bronchi, it could not be classified as a ductus arteriosus or bronchial artery. Therefore, a partially regressed isolated MAPCA with an aneurysm was diagnosed.

We believe that a symptomatic isolated MAPCA should be treated. Catheter intervention for isolated MAPCA in patients with pulmonary hypertension and left ventricular volume overload has been previously reported [3,6] as has catheter placement of a vascular plug to treat a ruptured MAPCA aneurysm in a patient with congenital heart disease who presented with hemoptysis [4]. However, to the best of our knowledge, treatment of an isolated blind-ended MAPCA with an aneurysm, as in our patient, has not been previously reported. Treatment was provided in this case because of the risks of aneurysm rupture and thrombus formation.

## Conclusions

In the case of abnormal vessels arising from the lesser curvature of the aortic arch and the echocardiogram not showing the details, contrast-enhanced computed tomography was useful in determining diagnosis and treatment. MAPCA can sometimes complicate the aneurysm, even when it has a blind end. Therapeutic intervention should be considered based on appropriate assessment.

## References

[1] Esparza-Hernández CN, Ramírez-González JM, Cuéllar-Lozano RA (2017). Morphological analysis of bronchial arteries and variants with computed tomography angiography. Biomed Res Int.

[2] Mainwaring RD, Patrick WL, Rosenblatt TR, Ma M, Kamra K, Arunamata A, Hanley FL (2019). Surgical results of unifocalization revision. J Thorac Cardiovasc Surg.

[3] Hoang LX, Tuyen LK, Gia TM (2023). Large isolated major aortopulmonary collateral artery causing dilated left ventricle. Radiol Case Rep.

[4] Sharma A, Kumar S, Priya S (2016). Ruptured aneurysm of major aortopulmonary collateral artery: management using Amplatzer vascular plug. Cardiovasc Diagn Ther.

[5] Alex A, Ayyappan A, Valakkada J, Kramadhari H, Sasikumar D, Menon S (2022). Major aortopulmonary collateral arteries. Radiol Cardiothorac Imaging.

[6] Harshith K, Anoop A, Jineesh V (2021). A rare cause of hemoptysis in West syndrome-isolated aortopulmonary collaterals in structurally normal heart. Indian J Radiol Imaging.

